# Cleavage Site Localization Differentially Controls Interleukin-6 Receptor Proteolysis by ADAM10 and ADAM17

**DOI:** 10.1038/srep25550

**Published:** 2016-05-06

**Authors:** Steffen Riethmueller, Johanna C. Ehlers, Juliane Lokau, Stefan Düsterhöft, Katharina Knittler, Gregor Dombrowsky, Joachim Grötzinger, Björn Rabe, Stefan Rose-John, Christoph Garbers

**Affiliations:** 1 Institute of Biochemistry, Kiel University, Kiel, Germany

## Abstract

Limited proteolysis of the Interleukin-6 Receptor (IL-6R) leads to the release of the IL-6R ectodomain. Binding of the cytokine IL-6 to the soluble IL-6R (sIL-6R) results in an agonistic IL-6/sIL-6R complex, which activates cells via gp130 irrespective of whether the cells express the IL-6R itself. This signaling pathway has been termed trans-signaling and is thought to mainly account for the pro-inflammatory properties of IL-6. A Disintegrin And Metalloprotease 10 (ADAM10) and ADAM17 are the major proteases that cleave the IL-6R. We have previously shown that deletion of a ten amino acid long stretch within the stalk region including the cleavage site prevents ADAM17-mediated cleavage, whereas the receptor retained its full biological activity. In the present study, we show that deletion of a triple serine (3S) motif (Ser-359 to Ser-361) adjacent to the cleavage site is sufficient to prevent IL-6R cleavage by ADAM17, but not ADAM10. We find that the impaired shedding is caused by the reduced distance between the cleavage site and the plasma membrane. Positioning of the cleavage site in greater distance towards the plasma membrane abrogates ADAM17-mediated shedding and reveals a novel cleavage site of ADAM10. Our findings underline functional differences in IL-6R proteolysis by ADAM10 and ADAM17.

The pleiotropic cytokine Interleukin-6 (IL-6) is an important mediator in nearly all physiological and patho-physiological conditions^1^,^2^. IL-6 activates downstream signaling pathways such as Janus kinase/Signal Transducer and Activator of Transcription (Jak/STAT), phosphatidyl-inositol-3-kinase (PI3K)-cascade and the mitogen activated protein kinase (MAPK)-cascade through a homodimer of glycoprotein 130 (gp130)^3^. To achieve specificity and to avoid unwanted cellular activation, IL-6 has to bind initially to the non-signaling alpha-receptor Interleukin 6-receptor (IL-6R), which shows a distinct expression pattern on only a limited number of cell types such as hepatocytes and some leukocytes^3^. This mode of signaling has been termed classic signaling and accounts mostly for the anti-inflammatory and regenerative functions of IL-6^4^,^5^.

The IL-6R is a type-I transmembrane protein with an extracellular part that consists of an Ig-like domain (‘D1’), two fibronectin-type-III domains (‘D2’ and ‘D3’), which build up the so-called cytokine-binding module (CBM), and a 55 amino-acid residues long flexible stalk region. Binding of IL-6 to the IL-6R induces the recruitment of two molecules gp130 and subsequent activation of the aforementioned signaling cascades. We have shown previously that the stalk region acts as a spacer that positions the D1 to D3 domains in a certain distance towards the plasma membrane^6^. A minimal length of 22 amino acids of the stalk region is required for efficient IL-6 classic signaling, which correspond to a stalk length of approximately 83 Å^6^.

Soluble forms of the IL-6R (sIL-6R) are predominantly generated by ADAM10- and ADAM17-mediated proteolysis^7^,^8^,^9^, and to a lesser extent by alternative splicing of the *IL6R* mRNA^10^. ADAM17-mediated shedding can be induced by several stimuli, e. g. the phorbol ester phorbol-12-myristate-13-acetate (PMA), which is the strongest known ADAM17 activator *in vitro*. Activation of ADAM10 can be achieved by cellular influx of Ca^2+^, e.g. via the ionophore ionomycin or ATP-mediated activation of purinergic P2 ×  7 receptors^7^. IL-6 binds to membrane-bound and soluble IL-6R with similar affinity, and the IL-6/sIL-6R complex can in principle activate all cells of the human body due to the ubiquitous expression of the signal-transducing β -receptor gp130. This so-called IL-6 trans-signaling is causative for the pro-inflammatory properties of IL-6, and its specific inhibition via soluble forms of gp130 has been shown to be beneficial in a variety of inflammatory animal models^11^.

The human IL-6R was shown to be cleaved by ADAM17 between Gln-357 and Asp-358 within the extracellular stalk region adjacent to the plasma membrane^12^. Consequently, deletion of the amino-acid residues Ser-353 to Val-362 resulted in an IL-6R variant that was resistant to ADAM17-mediated shedding, albeit it retained full biological activity^6^,^12^. This variant could still be shed by ADAM10, suggesting that either ADAM10 uses a different cleavage site than ADAM17, or that ADAM10 can cleave the IL-6R at multiple sites within the stalk region^6^. Although the cleavage site is a major determinant of substrate specificity, exosites have been proposed to contribute to substrate/protease interaction and subsequent shedding of the substrate. One example is CANDIS, a small juxtamembrane segment of ADAM17, which binds to the stalk of the IL-6R and modulates proteolysis of the receptor^13^.

Recently, a palindromic sequence containing three serine residues has been identified in the ADAM17 substrates CD163 and TNFα , and has been shown to be critical for ADAM17-mediated proteolysis of the two proteins^14^. In the present study, we identify a similar triple serine (3S) motif (Ser-359 to Ser-361) adjacent to the cleavage site within the IL-6R. We show that deletion of this motif prevents IL-6R cleavage by ADAM17. This deletion reduces the distance between the cleavage site and the plasma membrane, which is sufficient to block ADAM17-, but not ADAM10-mediated shedding. We further position the cleavage site in greater distance to the plasma membrane and characterize ADAM10- and ADAM17-mediated proteolysis of these variants. Finally, we show that ADAM10 and ADAM17 have non-redundant roles in IL-6R shedding in human cells.

## Results

### A triple serine (3S) motif is present adjacent to the ADAM17 cleavage site within the IL-6R

**Figure 1:**
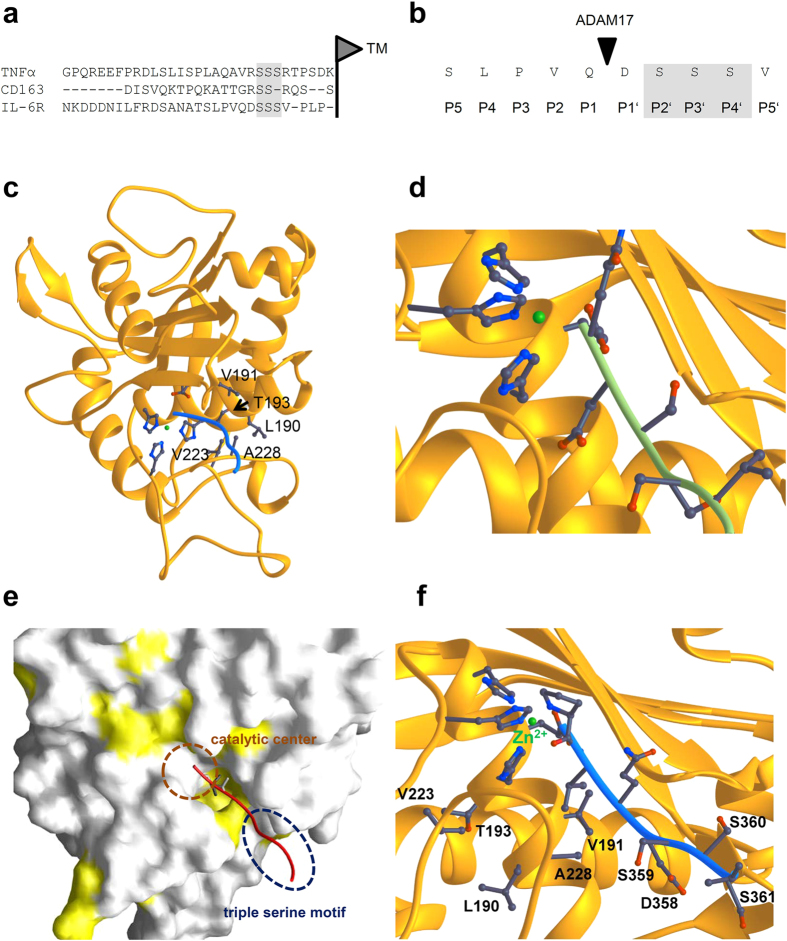
The IL-6R stalk region contains a 3S motif adjacent to the cleavage site. (**a**) Sequence alignment of the juxtamembrane regions of TNFα , CD163 (according to^14^) and the IL-6R. The serine-rich motifs within the three proteins are indicated by gray background color, and the start of the transmembrane regions is indicated by the vertical black line. (**b**) The five non-prime and prime sites of the ADAM17 cleavage site within the IL-6R. The 3S motif is highlighted by the gray background. (**c**) Based on the known structure of the catalytic domain of ADAM17 in complex with TIMP3, a model of the catalytic domain (orange) in complex with the corresponding amino-acid residues of the IL-6R stalk region (colored in blue) was generated. Critical amino acids of ADAM17 are given. (**d**) Close-up of the catalytic center of ADAM17. The IL-6R peptide QDSSSVP is shown in green. (**e**) Hydrophobic patches on the surface of the catalytic domain of ADAM17 are colored in yellow. The IL-6R peptide is shown in red. (**f**) Close-up of the catalytic center of ADAM17. The IL-6R peptide PVQDSSS is shown in blue.

ADAM10 and ADAM17 are the major proteases that generate the soluble IL-6R^7^,^15^, but how these proteolytic enzymes recognize their substrates is incompletely understood. Recently, a palindromic sequence (Arg-Ser-Ser-Ser-Arg) has been identified in the juxtamembrane stalk region in close proximity to the cleavage site of the prototypic ADAM17 substrate TNFα 14. Interestingly, also the membrane proximal stalk of CD163, another ADAM17 substrate, contains a comparable palindromic sequence (Arg-Ser-Ser-Arg)^14^,^16^. This prompted us to investigate whether such a sequence is also present within the IL-6R. As shown in the alignment of juxtamembrane regions of TNFα , CD163 and IL-6R, a triple serine (3S) motif is indeed located within the stalk region of the human IL-6R (Ser-359 to Ser-361), while there is no homology between the other parts of the stalk regions of the three proteins (Fig. 1a). In contrast to TNFα and CD163, it is not flanked by arginine residues, but it resides in a comparable distance to the plasma membrane (Fig. 1a). Importantly, this motif is located in close proximity to the described ADAM17 cleavage site between Gln-357 and Asp-358 and constitutes the prime sites P2’, P3’ and P4’ (amino-acid residues located C-terminal from the cleavage site) (Fig. 1b).

In order to elucidate the interaction of the IL-6R and ADAM17 and to explore a possible influence of the 3S motif on ADAM17-mediated proteolysis, we build a structural model of seven amino-acid residues of the stalk region including the cleavage site (QDSSSVP) bound to the active site of the catalytic domain of ADAM17 (Fig. 1c,d). Gln-357 and Asp-358 in combination with the 3S motif form a hydrophilic region within the IL-6R stalk, and deletion of the 3S motif would thus probably reduce interaction of the IL-6R with the hydrophilic environment of the catalytic domain of ADAM17 (Fig. 1e). Therefore, loss of the hydrophilic region within the IL-6R stalk could cause an impaired binding to the catalytic domain and impaired proteolysis of the IL-6R by ADAM17.

Surprisingly, modeling of the IL-6R peptide revealed that cleavage by ADAM17 between Gln-357 and Asp-358 is rather unlikely. Instead, the model suggests a cleavage of the IL-6R between Pro-355 and Val-356 when these two amino-acid residues were included into the model (PVQDSSS, Fig. 1f). The catalytic domain of ADAM17 forms a hydrophobic cavity adjacent to the catalytic center which could easily bind Pro-355/Val-356, but not Gln-357/Asp-358 (Fig. 1f). Indeed, a recent study showed that recombinant ADAM17 cleaved an IL-6R peptide, which consisted of the amino-acid residues Asp-347 to Thr-365 of the IL-6R stalk, between Pro-355/Val-356, which fits to our model^17^. Irrespective of this, the model suggests that deletion or mutation of the 3S motif within the IL-6R would not directly block interaction with the catalytic domain of ADAM17, but rather influence binding of the substrate to the protease.

### Deletion of the 3S motif abrogates ADAM17-mediated shedding of the IL-6R

To investigate the role of the 3S motif in ADAM17-mediated shedding, we transiently transfected HEK293 cells with cDNAs encoding either IL-6R wildtype or an IL-6R variant lacking the three serine residues (IL-6RΔ S359_S361). As a negative control, we transfected HEK293 cells with IL-6RΔ S353_V362, which has been shown to be resistant towards shedding by ADAM17^6^,^12^.

**Figure 2:**
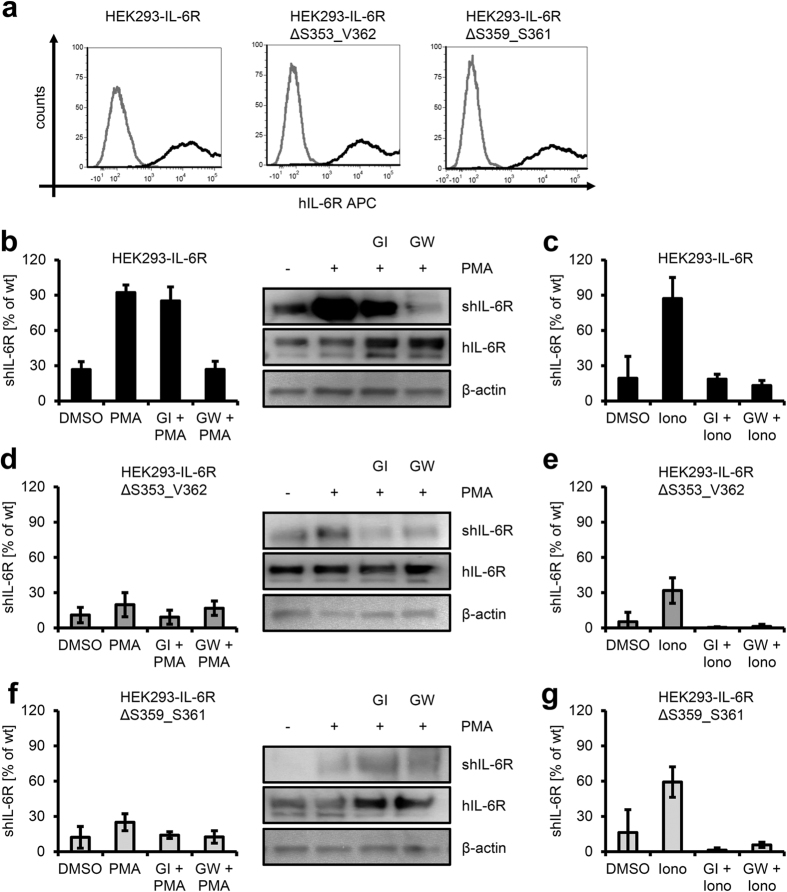
Deletion of the 3S motif is critical for ADAM17 mediated proteolysis. (**a**) HEK293 cells were transfected with expression plasmids encoding either wildtype human IL-6R, human IL-6R∆ S353_V362 and human IL-6R∆ S359_S361. 48 h later, cell surface expression of the three IL-6R variants was determined via flow cytometry. (**b**) HEK293 cells were transfected with an expression plasmid encoding wildtype human IL-6R. 48 h later, cells were treated for 2 h with PMA (100 nM) or DMSO as negative control. Cells were pre-treated with GI or GW 30 min prior to PMA stimulation where indicated. Generation of the soluble IL-6R was determined via ELISA or sIL-6R was precipitated from the cell culture supernatant and visualized via Western blotting. Cells were lysed and IL-6R expression determined via Western blotting. β -actin served as loading control. (**c**) HEK293 cells were transfected with an expression plasmid encoding wildtype human IL-6R. 48 h later, cells were treated for 1 h with ionomycin (1 μ M) or DMSO as negative control. Cells were pre-treated with 3 μ M GI or 3 μ M GW 30 min prior to ionomycin stimulation where indicated. Generation of the soluble IL-6R was determined via ELISA. (**d,e**) The experiment was performed as described under panels (b,c), but cells were transfected with pcDNA3.1-IL-6R∆ S353_V362. (**f,g**) The experiment was performed as described under panels (b,c), but cells were transfected with pcDNA3.1-IL-6R∆ S359_S361. ELISA data are the mean (± S.D.) of three independent experiments, Western blots and flow cytometry data show one representative experiment out of three performed.

First, we ensured that all three variants were equally well expressed at the cell surface. Flow cytometry revealed robust cell-surface expression of all three IL-6R variants at comparable levels (Fig. 2a). This indicates that the 3S motif is not necessary for intracellular sorting and transport to the cell surface, which is in agreement with previous results showing that even larger deletions of the stalk region do not prevent cell-surface presentation of the IL-6R^6^.

After confirming equal cell surface expression, we analyzed ADAM17-mediated proteolysis of the three IL-6R variants. We stimulated transiently transfected HEK293 cells for 2 h with the strong ADAM17 activator PMA. As shown in Fig. 2b, quantification of sIL-6R in the cell supernatant by ELISA revealed a strong increase of sIL-6R levels after PMA treatment. The same was observed when sIL-6R was precipitated from cell supernatant and detected via Western blotting (Fig. 2b). Pre-treatment of the cells with the ADAM10/ADAM17-specific inhibitor GW prevented PMA-induced proteolysis of the IL-6R, whereas the ADAM10-specific inhibitor GI showed no significant effect (Fig. 2b), confirming previous results that PMA induced sIL-6R generation in an ADAM17-dependent manner^6^,^7^,^9^,^12^.

Stimulation of cells with the ionophore ionomycin activates ADAM10-mediated IL-6R shedding^6^,^7^,^18^. We recapitulated these findings in HEK293 cells transiently transfected with an IL-6R wildtype cDNA (Fig. 2c). Indeed, ionomycin treatment enhanced sIL-6R generation, which could be blocked equally well with GI or GW (Fig. 2c).

ADAM17 cleaves the IL-6R between Gln-357 and Asp-358 within the stalk region in close proximity to the plasma membrane^12^, and consequently a ten amino acid long deletion spanning Ser-353 to Val-362 has been shown to be resistant to proteolysis by ADAM17^6^,^12^. To compare shedding of different IL-6R variants, we set the amount of sIL-6R generated after PMA stimulation of HEK293 cells transfected with wild-type IL-6R to 100% (Figs 2b and 6) and calculated all other values accordingly. Indeed, PMA-treatment of IL-6RΔ S353_V362 transfected cells resulted in strongly reduced sIL-6R generation of only 19.7 ±  10.3% compared to wild-type IL-6R (Fig. 2d). Pre-treatment with the metalloprotease inhibitors GI or GW further reduced sIL-6R generation, showing that the IL-6R variant could still be shed constitutively by ADAM10 (Fig. 2d). In line with this, forced activation of ADAM10 enhanced sIL-6R generation^6^ (and Fig. 2e).

To determine a possible role of the 3S motif in proteolysis of the IL-6R by ADAM17, we transiently transfected HEK293 cells with a cDNA encoding IL-6RΔ S359_S361. Treatment of the cells with PMA resulted in only 25.0 ±  7.2% sIL-6R generation compared to wild-type IL-6R (Fig. 2f). As seen for the IL-6RΔ S353_V362 variant, pre-treatment with GI or GW only slightly reduced IL-6R shedding (Fig. 2f). We verified this observation via Western blotting, and observed only a minimal increase in sIL-6R generation after PMA treatment (Fig. 2f). In contrast, the IL-6RΔ S353_V362 variant was still shed by ADAM10, although in a slightly reduced manner compared to wildtype IL-6R (59.2 ±  13.0%, Fig. 2g), underlining once more fundamental differences in ADAM10- and ADAM17-mediated proteolysis of the IL-6R.

In conclusion, these data show that deletion of the 3S motif Ser-359 to Ser-361 is as effective in preventing ADAM17-, but not ADAM10-mediated, proteolysis of the IL-6R as deletion of the larger amino acid stretch Ser-353 to Val-362 which contains the ADAM17 cleavage site. This suggests that the 3S motif might be critically involved in ADAM17 recognition and/or proteolysis of the IL-6R.

### Mutation of the 3S motif does not influence shedding by ADAM17 or ADAM10

Deletion of the 3S motif not only disrupts the hydrophilic interaction between IL-6R and ADAM17 (Fig. 1e), but also reduces the distance between the cleavage site Gln-357/Asp-358 and the plasma membrane. The cleavage site is separated from the membrane by six amino-acid residues, which span 22.8 Å (3.8 Å per amino-acid residue). The observed reduction in proteolysis of IL-6RΔ S359_S361 might therefore either be a direct effect of the 3S motif, or be due to sterical hindrance between ADAM17 and its substrate.

In order to investigate this further, we replaced the 3S motif with either three alanine residues (IL-6R_AAA), or a glutamic acid followed by two lysine residues (IL-6R_EKK). Tucher *et al.* performed cleavage site profiling of ADAM17 with the help of peptide libraries and showed that glutamic acid and lysine residues are disfavored at the prime sites P3’, P4’ and P5’ of ADAM17 cleavage sites^19^. Thus, a direct effect of the 3S motif would be lost in the IL-6R_EKK mutant.

**Figure 3:**
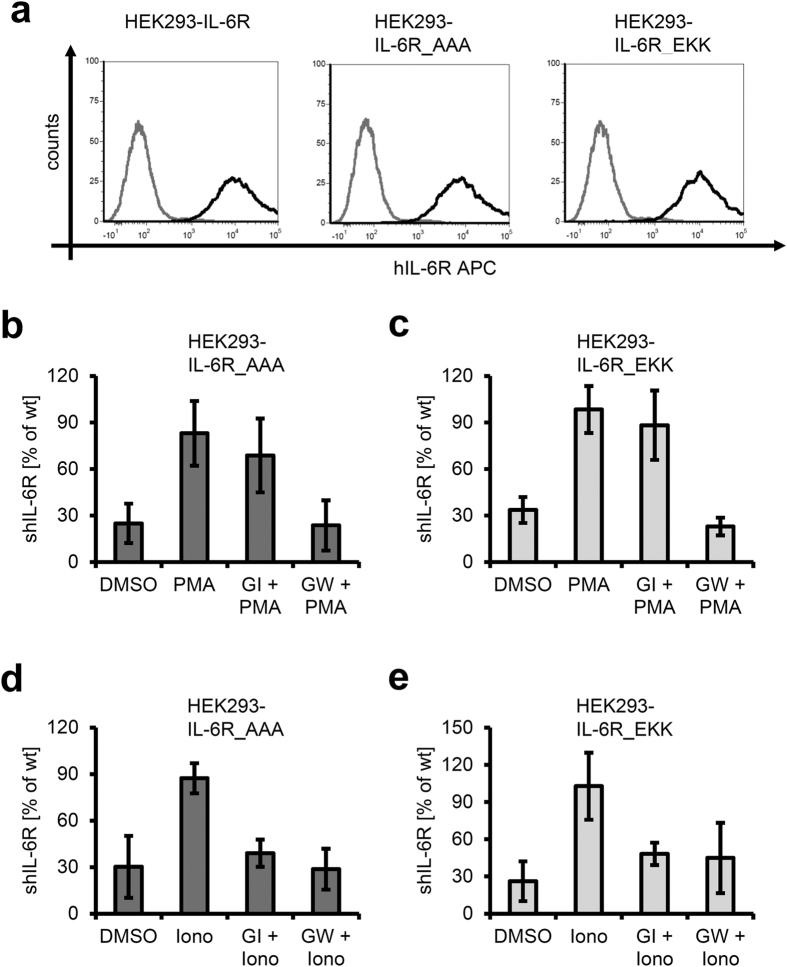
Substitution of the 3S motif only marginally influences proteolysis by ADAM17. (**a**) HEK293 cells were transfected with expression plasmids encoding either wildtype human IL-6R, human IL-6R_AAA or human IL-6R_EKK. 48 h later, cell surface expression of the three IL-6R variants was determined via flow cytometry. (**b**) HEK293 cells were transfected with an expression plasmid encoding human IL-6R_AAA. 48 h later, cells were treated for 2 h with PMA (100 nM) or DMSO as negative control. Cells were pre-treated with GI or GW 30 min prior to PMA stimulation where indicated. Generation of the soluble IL-6R was determined via ELISA. (**c**) The experiment was performed as described under panel (b), but cells were transfected with pcDNA3.1-IL-6R_EKK. (**d**) HEK293 cells were transfected with an expression plasmid encoding human IL-6R_AAA. 48 hours later, cells were treated for 1 h with ionomycin (1 μ M) or DMSO as negative control. Cells were pre-treated with 3 μ M GI or 3 μ M GW 30 min prior to ionomycin stimulation where indicated. Generation of the soluble IL-6R was determined via ELISA. (**e**) The experiment was performed as described under panel (**d**), but cells were transfected with pcDNA3.1-IL-6R_EKK. ELISA data are the mean (± S.D.) of three independent experiments. Flow cytometry data show one representative experiment out of three performed.

We transiently transfected HEK293 cells with cDNAs encoding both IL-6R variants and verified equal expression at the cell surface via flow cytometry (Fig. 3a). PMA-induced proteolysis was basically unaltered in both IL-6R_AAA (82.96 ±  20.92% compared to wildtype IL-6R, Fig. 3b) and IL-6R_EKK (98.33 ±  15.21%, Fig. 3c). Again, only pre-treatment with GW prevented shedding of the IL-6R (Fig. 3b,c). We observed the same when we stimulated both variants with ionomycin to study proteolysis by ADAM10 (87.32 ±  9.72% and 102.74 ±  27.09%, respectively, Fig. 3d,e).

These results suggest that the impaired proteolysis of IL-6RΔ S359_S361 is not a direct cause due to the loss of the 3S motif, but rather originates from the shortened distance between the cleavage site and the plasma membrane.

### N-terminal displacement of the cleavage site blocks ADAM17- and alters ADAM10-mediated proteolysis

**Figure 4:**
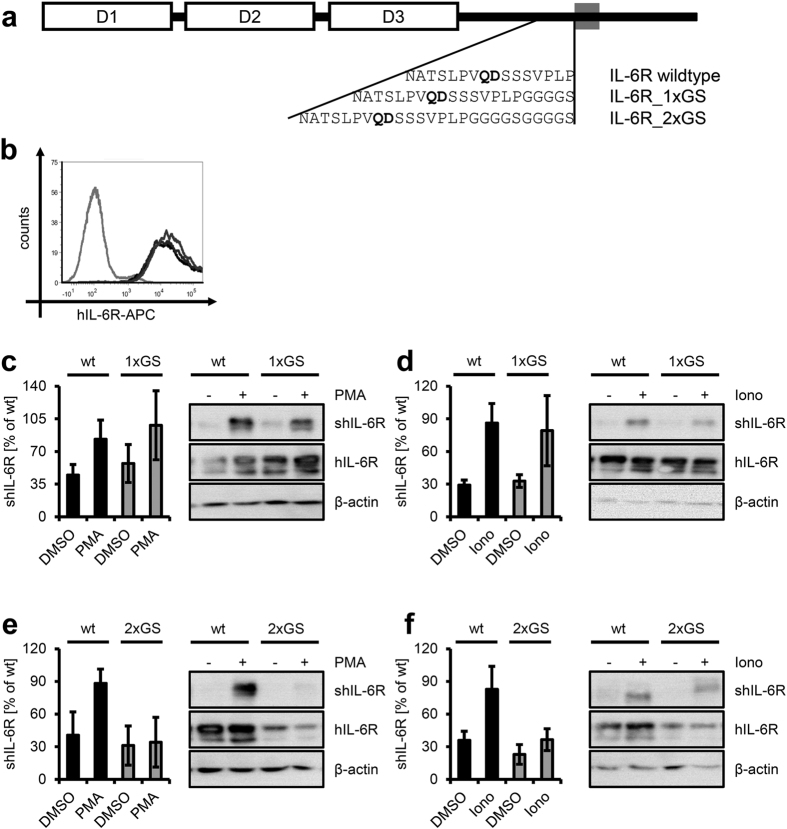
Juxtamembrane elongation of the IL-6R stalk differentially influences proteolysis by ADAM10 and ADAM17. (**a**) Schematic representation of the IL-6R. The Ig-like domain (‘D1’) is followed by two fibronectin-type-III domains (‘D2’ and ‘D3’), a flexible stalk region, the transmembrane region and the intracellular region. The juxtamembrane amino acid sequences of the wildtype IL-6R and the prolonged variants IL-6R_1xGS and IL-6R_2xGS are shown below. The ADAM17 cleavage site is indicated in bold. (**b**) HEK293 cells were transfected with expression plasmids encoding wildtype IL-6R, IL-6R_1xGS and IL-6R_2xGS. 48 h later, cell surface expression of the IL-6R variants was determined via flow cytometry: negative control (light gray), wildtype IL-6R (black), IL-6R_1xGS (dark gray), IL-6R_2xGS (gray). (**c**) HEK293 cells were transfected with expression plasmids encoding either wildtype human IL-6R or IL-6R_1xGS. 48 h later, cells were treated for 2 h with PMA (100 nM) or DMSO as negative control. Generation of the soluble IL-6R was determined via ELISA or sIL-6R was precipitated from the cell supernatant and visualized via Western blotting. Cells were lysed and IL-6R expression determined via Western blotting. β -actin served as loading control. (**d**) The experiment was performed as described under panel (**c**), but cells were treated for 1 h with Ionomycin (1 μ M) or DMSO as negative control. (**e,f**) The experiments were performed as described under panels (c,d), but cells were transfected with either wildtype human IL-6R or IL-6R_2xGS. ELISA data are the mean (± S.D.) of three independent experiments, Western blots and flow cytometry data show one representative experiment out of three performed.

Having shown that reduction of the distance between the cleavage site and the plasma membrane affects ADAM17-, but not ADAM10-mediated shedding of the IL-6R, we sought to investigate how an increased distance would affect IL-6R proteolysis. To achieve this, we generated two IL-6R variants in which either one or two GGGGS linker motifs were placed at the C-terminus of the stalk region (IL-6R_1xGS and IL-6R_2xGS, Fig. 4a). Each of these juxtamembrane extensions enhances the distance of the cleavage site and the plasma membrane by 19 Å, a total distance of 41.8 Å (IL-6R_1xGS) and 60.8 Å (IL-6R_2xGS). This strategy has the advantage that all regulatory elements, like the CANDIS binding region^13^, remain within the same distance to each other, as it just moves the whole extracellular part of the IL-6R further away from the plasma membrane. In contrast, moving just the cleavage site inside of the stalk would probably cause additional effects with regard to IL-6R/ADAM17 interaction. Furthermore, glycine and serine residues are disfavored within ADAM17 cleavage sites^19^, thus reducing the chance to introduce novel artificial cleavage sites.

Like before, we transiently transfected HEK293 cells with cDNAs encoding both IL-6R variants. Elongation of the stalk region did not influence equal expression at the cell surface, as detected via flow cytometry (Fig. 4b). When we stimulated the cells with PMA, we could not detect a difference of the IL-6R_1xGS variant in comparison to wildtype IL-6R via ELISA (98.05 ±  37.02%, Fig. 4c) or via Western blot of the precipitated supernatant (Fig. 4c). We observed no reduction in ionomycin-induced ADAM10-mediated proteolysis of the IL-6R_1xGS variant (79.15 ±  32.31%, Fig. 4d). Western blot of the precipitated receptors from the supernatant also revealed no significant difference compared to the wildtype IL-6R (Fig. 4d). Importantly, the cleavage products of the IL-6R_1xGS variants had the same size as the cleaved wildtype IL-6R, indicating that the two proteases still cleave at the original cleavage site (Fig. 4c,d). However, small changes of a few amino acid residues might not be detected by Western blotting. We concluded that shifting of the cleavage site by five amino-acid residues away from the plasma membrane does not impede proteolysis of the IL-6R by ADAM10 or ADAM17.

We assessed cleavage of the IL-6R_2xGS variant by the same experimental strategy. As shown in Fig. 4e, stimulation with PMA resulted in impaired cleavage of IL-6R_2xGS (34.19 ±  22.91%), which did not differ from vehicle treated cells (31.13 ±  18.02%). Likewise, we detected only a faint band at the molecular size of the cleaved IL-6R via Western blot after PMA stimulation (Fig. 4e). Although the IL-6R_2xGS variant appeared to be less expressed compared to wildtype IL-6R in total cell lysates (Fig. 4e), this could not solely account for the reduced proteolysis, because the amount at the cell surface was not reduced, where proteolysis by ADAM10 and ADAM17 takes place (Fig. 4b). In contrast, shedding via ADAM10 was still inducible (36.56 ±  10.02% vs. 22.9 ±  9.05%), albeit to a much lower extend as the wildtype IL-6R (Fig. 4f). Surprisingly, analysis of the precipitated sIL-6R revealed an increased molecular weight of the cleavage product of the IL-6R_2xGS variant, indicating that ADAM10 did not use the original IL-6R cleavage site, but rather cleaved at a more C-terminal site, probably even within the GGGGS linker (Fig. 4f). However, cleavage profiling of ADAM10 did not show preferences for glycine or serine residues at P1 or P1’ positions^19^.

Collectively, these data show that both ADAM10 and ADAM17 cannot cleave the IL-6R at their original cleavage site when it is located in a certain distance from the cell membrane, and that the critical distance lies between 41.8 Å and 60.8 Å.

### Shortening of the IL-6R stalk does not block IL-6 classic signaling

Binding of IL-6 to its receptor induces recruitment of two molecules of the β -receptor gp130 and subsequent activation of intracellular signaling cascades, mainly the Jak/STAT pathway^20^,^21^. To investigate whether deletion of the 3S motif influences IL-6 classic signaling, we stably transduced Ba/F3-gp130 cells with either wild-type IL-6R or the mutated IL-6R variants. Ba/F3 cells are murine pre-B cells, which proliferate in strict dependence on IL-3 stimulation. Stable expression of gp130 renders the cells responsive towards stimulation on IL-6 in combination with the soluble IL-6R (sIL-6R) or Hyper-IL-6, which is a fusion protein of IL-6/sIL-6R^22^. As shown previously, a second transduction with IL-6R cDNA results in Ba/F3-gp130-IL-6R cells which proliferate in response to IL-6 without the need of the sIL-6R^6^,^23^.

**Figure 5:**
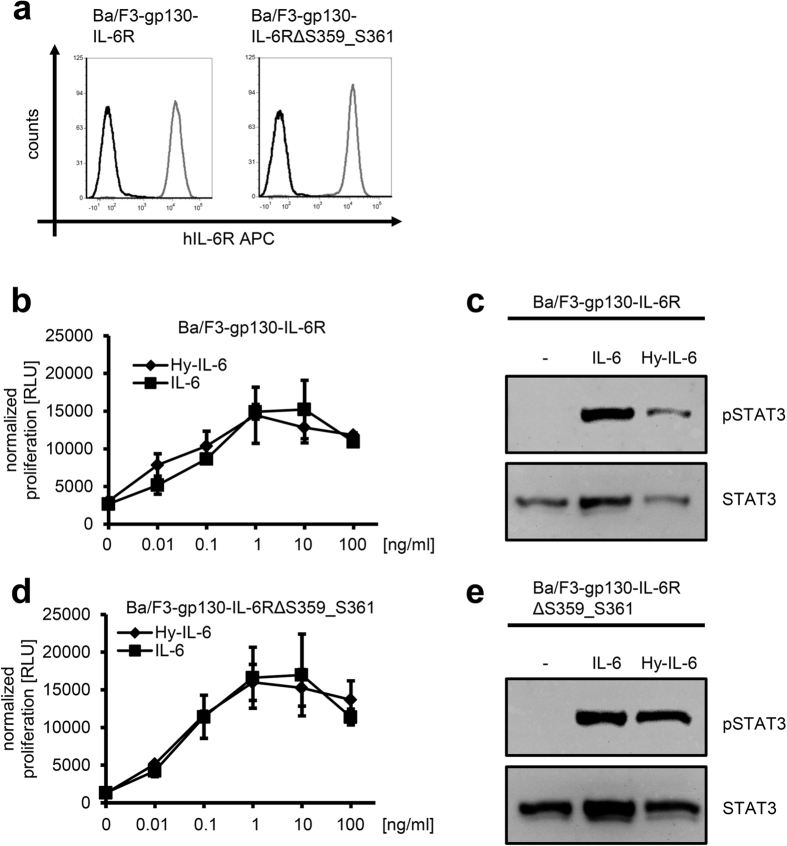
Modification of the stalk region does not influence the biological activity of the IL-6R. (**a**) Cell surface expression of IL-6R on stably transduced Ba/F3-gp130-IL-6R and Ba/F3-gp130-IL-6R∆ S359_S361 cells was determined via flow cytometry. (**b**) Equal amounts of Ba/F3-gp130-IL-6R cells were incubated for 48 h with the indicated concentration of either IL-6 or Hyper-IL-6. Cell proliferation was determined as described in *Experimental Procedures*. (**c**) Equal amounts of Ba/F3-gp130-IL-6R cells were starved for 2 h in serum free medium and stimulated with 10 ng/ml IL-6 or 10 ng/ml Hyper-IL-6 for 15 min or left untreated. Phosphorylation of STAT3 was determined by Western blotting. Total STAT3 served as endogenous loading control. (**d,e**) The experiments were performed as described under panels (c,d), but with stably transduced Ba/F3-gp130-IL-6R∆ S359_S361 cells. Data show one representative experiment out of three performed.

First, we verified equal cell-surface expression of all IL-6R variants on the stably transduced Ba/F3-gp130 cells (Fig. 5a). Ba/F3-gp130-IL-6R cells proliferated in a concentration-dependent manner in response to both IL-6 and Hyper-IL-6, and both cytokines induced activation of intracellular signaling cascades as visualized by phosphorylation of the transcription factor STAT3 (Fig. 5b,c). We have previously shown that IL-6R mutants with shortened stalks proliferate in response to IL-6 classic signaling as long as the stalk region comprises 22 amino-acid residues^6^. Accordingly, Ba/F3-gp130-IL-6RΔ S359_S361 cells proliferated equally well in a dose-dependent manner when stimulated with increasing amounts (0–100 ng/ml each) of either IL-6 or Hyper-IL-6 (Fig. 5d). Similarly, short-term treatment of the cell lines for 15 min with either IL-6 or Hyper-IL-6 resulted in robust STAT3 phosphorylation, showing that the truncated IL-6R IL-6RΔ S359_S361 is fully biologically active (Fig. 5e).

### Genetic depletion of ADAM10 and ADAM17 confirms non-redundant roles in proteolysis of the human IL-6R

**Figure 6:**
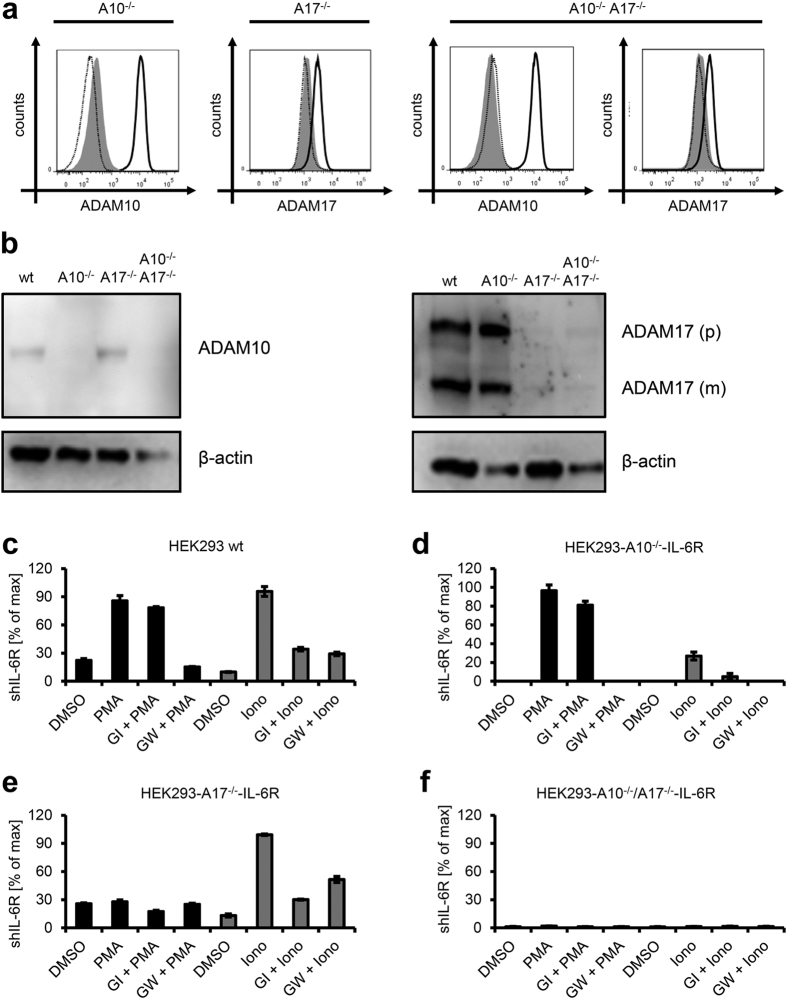
ADAM10 and ADAM17 are the major IL-6R sheddases. (**a**) Cell-surface expression of ADAM10 and ADAM17 on CRISPR/Cas9-generated single and double knock-out HEK293 cells. Protease expression on the parental HEK293 cells is indicated by the black line, whereas protease expression of the respective knock-out cell line is shown by a dashed line. The isotype control is shown as a filled gray histogram. (**b**) Absence of ADAM10 in lysates of ADAM10 (A10^−/−^) and A10^−/−^/A17^−/−^ cells and absence of ADAM17 in A17^−/−^ and A10^−/−^/A17^−/−^ cells was confirmed by Western blotting. β -actin served as loading control. (**c–e**) The indicated HEK293 knock-out cell lines were transiently transfected with wildtype IL-6R. Cells were stimulated 48 h later as described in the legend of Fig. 2 and sIL-6R was quantified via ELISA. In each panel, one representative experiment out of three performed is shown.

ADAM10 and ADAM17 can be selectively activated by ionomycin and PMA^7^,^15^. However, ionomycin-induced compensatory shedding in ADAM10^−/−^ murine embryonic fibroblasts (MEFs) by ADAM17 has been shown for the IL-6R^7^ and other substrates^24^. Furthermore, sIL-6R was still present in experiments with ADAM10^−/−^/ADAM17^−/−^ MEFs, suggesting the existence of another protease that is capable to shed the IL-6R^7^. To clarify these issues in a human setting, we created HEK293 cells deficient in ADAM10 (HEK293-A10^−/−^), ADAM17 (HEK293-A17^−/−^) or both proteases (HEK293-A10^−/−^/A17^−/−^) via the CRISPR/Cas9-system. We verified the successful deletion of the two proteases via flow cytometry (Fig. 6a) and Western blot of total cell lysates (Fig. 6b).

Whereas both PMA and ionomycin induced proteolysis in wildtype HEK293 cells (Fig. 6c), only PMA-inducible shedding of the IL-6R was unaltered in HEK293-A10^−/−^ cells (Fig. 6d). In contrast, proteolysis after ionomycin-stimulation was drastically reduced and even further impaired when cells were pre-treated with the ADAM10-inhibior GI (Fig. 6d). Thus, in contrast to ADAM10^−/−^ MEFs, ADAM17 could not be activated by ionomycin and was not able to act as a compensatory sheddase in the human system. Ionomycin-induced shedding was intact, however, when we investigated IL-6R proteolysis in HEK293-A17^−/−^ cells, whereas stimulation with PMA had no effect (Fig. 6e). Genetic deletion of both proteases revealed no inducible shedding by either PMA or ionomycin, and the total levels of sIL-6R generated by these cells were between 1 and 2% compared to sIL-6R levels generated after PMA stimulation in HEK293 wildtype cells (Fig. 6f).

Thus, it appears that no other protease exists within this human cell system that efficiently cleaves the IL-6R. However, one publication showed that cathepsin G, a neutrophil serine protease, can release sIL-6R from cells^25^, and we cannot rule out that other proteases, that are not expressed in HEK293 cells, can cleave the human IL-6R in other cells.

## Discussion

ADAM10 and ADAM17 are important catalytic enzymes in health and disease. Some substrates are only cleaved by ADAM10, some only by ADAM17, and others are cleaved by both proteases. It is still under debate how these proteases distinguish between substrates and non-substrates. Several structural traits, on both the proteases and their substrates, have been identified to contribute to specific substrate-protease interaction.

There are three major findings in the present study. First, we identify a 3S motif within the IL-6R stalk region that is reminiscent of serine-rich motifs that have been identified in the ADAM17 substrates TNFα and CD163. Second, we show that deletion of these three amino-acid residues does not block shedding by ADAM10, but is sufficient to prevent cleavage of the IL-6R by ADAM17. This impaired IL-6R processing is caused by the shortened distance between the cleavage site and the plasma membrane. Third, we demonstrate that elongation of the stalk region, by positioning the cleavage site in a greater distance to the plasma membrane, blocks proteolysis by ADAM17, and leads to cleavage by ADAM10 at a novel cleavage site.

Interestingly, albeit more than 100 substrates have been identified that are cleaved by either ADAM10 or ADAM17 alone or by both proteases, it has been extremely challenging to identify a consensus cleavage sequence. Nevertheless, all substrates in which the cleavage site has been determined are shed in close proximity to the plasma membrane. We have previously reported that deletion of ten amino-acid residues (Ser-353 to Val-362) results in an IL-6R variant that cannot be cleaved by ADAM17, which is expected since it contains the reported cleavage site Gln-357/Asp-358^12^. Shedding of a further variant of the IL-6R (IL-6RΔ D358_V362), the deletion of which included the 3S motif, was also greatly impaired^12^. An IL-6R variant lacking 35 amino-acid residues within the stalk region (IL-6RΔ E317_T352) showed only 4% sIL-6R generation by ADAM17 as compared to wildtype IL-6R, although the cleavage site was unchanged^6^. This highlights the possibility that besides the cleavage site other regions of the IL-6R must be recognized by ADAM17 to ensure proper proteolysis.

In this study, we show that even a smaller deletion is sufficient to prevent proteolysis of the IL-6R. The 3S motif is located in close proximity to Gln-357/Asp-358 on prime sites P2’, P3’ and P4’ C-terminal from the cleavage site. Although deletion of Ser-359 to Ser-361 left the cleavage site intact, it efficiently prevented proteolytic processing of the receptor, suggesting a pivotal role of prime site amino-acid residues in substrate recognition by ADAM17, but not ADAM10. Importantly, the deletion did not affect the ability of the IL-6R to initiate signal transduction after activation by IL-6.

However, the deletion moves the cleavage site closer to the plasma membrane and reduces the distance from 22.8 Å to 11.4 Å. In order to distinguish between sterical hindrance and a direct effect of the 3S motif, we exchanged the serine residues with amino-acid residues that are disfavored by ADAM17. Mutagenesis revealed unaltered shedding of the IL-6R by ADAM17, suggesting that indeed the positioning of the cleavage site closer to the plasma membrane caused the inhibitory effect. This is in clear contrast to the data of the 3S motif in the other ADAM17 substrates TNFα and CD163, where mutations within the 3S motif were sufficient to prevent ADAM17-mediated proteolysis^14^, and indicates that serine-rich motifs in close proximity to the cleavage site might have different functions and effects concerning proteolysis by ADAM17. Importantly, ADAM10 was still able to shed IL-6RΔ S359_S361, underlining another difference in the mode of action of these two proteases.

Surprisingly, our molecular modeling approach suggests that ADAM17 might prefer a cleavage site within the IL-6R located between Pro-355 and Val-356 instead of Gln-357 and Asp-358. Indeed, the IL-6R is the only ADAM17 substrate that is cleaved between a glutamine and an aspartic acid according to the MEROPS database^26^. If ADAM17 would indeed cleave between Pro-355 and Val-356, two serine residues of the 3S motif would be located outside of the cleavage site, which could explain why mutations of the serine residues do not block proteolysis. However, further experiments have to be performed to verify which cleavage site is actually used by ADAM17 *in vitro* and *in vivo*.

Insertion of small linker peptides between the stalk and the transmembrane regions of the IL-6R allowed for the first time to analyze if ADAM10 and ADAM17 can also cleave the IL-6R in larger distance to the plasma membrane. Addition of five amino-acid residues, thus enlarging the distance between membrane and cleavage site from 22.8 Å to 41.8 Å, did not alter shedding by ADAM10 or ADAM17, indicating that both proteases are able to access cleavage sites of their substrates within a certain range. However, further incresase to 60.8 Å prevented ADAM17-mediated proteolysis, indicating that a limit exists within which the cleavage site has to be localized. Importantly, ADAM10 was still able to cleave the IL-6R variant, although the size of the cleaved IL-6R indicates that ADAM10 uses a novel cleavage site further downstream and not its original cleavage site.

Analysis of human IL-6R shedding relies often on the use of chemical inhibitors or protease-deficient murine cells. Interestingly, shedding in ADAM10^−/−^ MEFs can be stimulated with ionomycin, although ADAM10 is missing, and calcium influx in normal cells only activates ADAM10, but not ADAM17^7^,^18^,^24^. Here, we show that this kind of compensatory shedding does not exist in human HEK293 cells devoid of ADAM10. Future work has to address which mechanisms underlie the compensatory shedding in ADAM10^−/−^ MEFs, and how ADAM17 can be activated by calcium influx in these cells. Release of the IL-6R into the supernatant was reduced to 1–2% in HEK293-A10^−/−^/A17^−/−^ cells, indicating that these two proteases release nearly all sIL-6R.

A further mechanism to generate soluble receptors is the cellular release of extracellular vesicles (EVs) from receptor-expressing cells^27^,^28^. Here, especially small vesicles derived from multivesicular bodies, called exosomes, are involved in numerous processes. Among the full-length proteins, which have been described to reside on EVs, are several substrates for ADAM10 and ADAM17, e.g. ICAM-1^29^, TNFR1^30^ and the IL-6R^31^. We have shown previously that ectodomain shedding as well as microvesicles contributes to the release of signaling molecules from cells which have undergone senescence^32^. Interestingly, also biologically active ADAM10 and ADAM17 have been found on EVs^33^, indicating that proteolytic cleavage of substrates from released microvesicles might also be possible.

In conclusion, we provide evidence that positioning of the cleavage site within the stalk region of the IL-6R is critical for proteolysis by ADAM17, and that deletion of three amino-acid residues C-terminal to the cleavage site is sufficient to block proteolysis. The cleavage site has to be located in a certain area to allow ADAM17-mediated proteolysis, whereas ADAM10 appears to be less restricted in this manner. Finally, experiments with protease-deficient human cells prove for the first time that ADAM10 and ADAM17 are the dominant proteases in human HEK293 cells that cleave the IL-6R.

## Methods

### Cells and Reagents

Ba/F3-gp130 cells were obtained from Immunex (Seattle, WA, USA^34^). HEK293 cells were from DSMZ GmbH (Braunschweig, Germany), and Phoenix-Eco cells from U. Klingmüller (DKFZ, Heidelberg, Germany). All cells were grown under standard conditions in DMEM high glucose culture medium (Sigma-Aldrich, Taufkirchen, Germany). DMEM was supplemented with 10% fetal bovine serum, penicillin (60 mg/l) and streptomycin (100 mg/l), and cells were kept at 37 °C and 5% CO_2_ in a standard incubator with a water-saturated atmosphere. Ba/F3-gp130 cells were cultured using 10 ng/ml of the recombinant IL-6/sIL-6R fusion protein Hyper-IL-6, which was expressed and purified according to^35^,^36^. After stable transduction (see below) with the different IL-6R variants, Ba/F3-gp130 were cultured with 10 ng/ml recombinant human IL-6, which was expressed and purified as described previously^37^. The anti-hIL-6R mAb 4-11 was described previously^38^. The anti-β -actin mAb, the anti-phospho STAT3 mAb (Tyr705) and anti-STAT3 mAb (124H6) were purchased from Cell Signaling Technology (Frankfurt/M., Germany). The antibody against ADAM10 (EPR5622) was from Gene Tex (Irvine, USA), the anti-ADAM17 antibody A300D has been described before^39^. The peroxidase conjugated secondary antibodies were obtained from Pierce (Thermo Scientific, Perbio, Bonn, Germany), and the APC-conjugated goat anti-mouse IgG was from Jackson ImmunoResearch Laboratories (Dianova GmbH, Hamburg, Germany). PMA (phorbol-12-myristate-13-acetate) and ionomycin were purchased from Sigma-Aldrich (Deisenhofen, Germany). The two metalloprotease inhibitors GW280264X (GW, selective for ADAM10 and ADAM17) and GI254023X (GI, selective for ADAM10) were synthesized by Iris Biotech (Marktredwitz, Germany)^40^,^41^.

### Construction of expression plasmids for the human IL-6R and deletion variants thereof

An expression plasmid encoding the human IL-6R in pcDNA3.1 has been described previously^7^, as well as the deletion variant pcDNA3.1-hIL-6RΔ S353_V362^6^. The novel IL-6R deletion variant pcDNA3.1-hIL-6RΔ S359_S361 was constructed via splicing by overlapping extension (SOE)-PCR and cloned into pcDNA3.1 via a 5’ KpnI site and a 3’ NotI site. The stalk variants pcDNA3.1-hIL-6R_1xGS and pcDNA3.1-hIL-6R_2xGS were created similarly by SOE-PCR. For retroviral transduction of Ba/F3-gp130 cells, the IL-6R constructs were subcloned into the pMOWS plasmid^42^.

### Retroviral transduction of Ba/F3-gp130 cells

Retroviral transduction of the murine pre-B cell line Ba/F3-gp130 via Phoenix-Eco cell supernatant has been described in detail previously^23^,^42^,^43^. Selection was achieved with puromycin (1.5 μ g/ml) and subsequent cultivation with 10 ng/ml recombinant IL-6.

### Generation of CRISPR/Cas9 plasmids

To specify the target region for the CRISPR/Cas9-mediated genome editing, the third exon of the human ADAM10 genomic sequence (ENST00000260408) and the first exon of the human ADAM17 genomic sequence (ENST00000310823) were submitted to an online CRISPR Design Tool (http://tools.genome-engineering.org). For each targeted gene, a pair of oligonucleotides (A10g#2_F: accGATACCTCTCATATTTACAC, A10g#2_R: aacGTGTAAATATGAGAGGTATC; A17g#2_F: accGCCGCGACCTCCGGATGACC, A17g#2_R: aacGGTCATCCGGAGGTCGCGGC) was annealed, phosphorylated and subsequently cloned into the *Sap*I-digested guideRNA expressing plasmid LeGO-Cas9-iC-puro +  cgRNA_SapI (generously provided by Boris Fehse, UKE Hamburg) according to the published protocol^44^. The resulting plasmids were designated LeGO-Cas9-ADAM10 and LeGO-Cas9-ADAM17, respectively.

### Genome-editing to achieve protease-deficient HEK293 cells

CRISPR/Cas9 plasmids were transfected into HEK293T cells using TurboFect Transfection Reagent (Life Technologies, Carlsbad, CA). 48 h following transfection, cells were grown in the presence of 1 μ g/ml puromycin for additional 48 h to enrich successfully transfected cells. To obtain ADAM10/ADAM17 double knock-out clones, LeGO-Cas9-ADAM10-transfected cells were subsequently transfected with LeGO-Cas9-ADAM17 two weeks following the first transfection. For the isolation of single genome-edited cells, cell populations were subjected to FACS (fluorescence activated cell sorting) analysis after immunostaining with PE anti-human CD156c antibody (BioLegend, San Diego, CA) and A300E antibody (Institute of Biochemistry, Kiel, Germany), respectively, or with both antibodies simultaneously, and single-cell sorted into 96-well plates. Single cell clones were further expanded and the efficient knock-out of ADAM10, ADAM17 or both proteases was confirmed via FACS and Western blotting.

### Ectodomain shedding assays in HEK293 cells

Transfection of HEK293 cells with different IL-6R variants and analysis of IL-6R ectodomain shedding by ADAM17 has been described elsewhere^6^,^7^.

### Precipitation of soluble IL-6R and Western blotting

The sIL-6R was precipitated by adding 100 μ l Concanavalin A beads (Sigma Aldrich) to 900 μ l of each cell culture supernatant. After incubation at 4 °C overnight under constant agitation, beads were washed 3 times with 1 ml PBS and boiled at 95 °C for 5 min in 25 μ l 3× Laemmli buffer (6% SDS, 30% Glycerol, 5% β -mercaptoethanol, 150 mM Tris-HCl (pH 6.8) and 0.2% bromphenol blue). Cells were lysed in mild lysis buffer (50 mM Tris, pH 7.5, 150 mM NaCl, 1% Triton X-100 and complete protease inhibitor mixture tablets (Roche Applied Science). Protein lysates and precipitated sIL-6R were separated by SDS-PAGE under reducing conditions and transferred to a PVDF membrane (Millipore, Darmstadt, Germany). Membranes were blocked with 5% skim milk powder in TBS-T (10 mM Tris-HCl, pH 7.6, 150 mM NaCl and 0.05% Tween 20). The membrane was probed with anti-hIL-6R (4–11) mAb at 4 °C overnight. After five washing steps with TBS-T, membranes were probed with an anti-mouse-antibody conjugated to horseradish peroxidase, and proteins were detected with West Femto Maximum Sensitvity Substrate Western Blotting Detection Kit (Thermo Scientific, Waltham, MA, USA) according to the manufacturer’s instructions. Afterwards, the membrane was washed 5 times with PBS-T and probed with an anti β -actin mAb (Cell Signaling). After five washing steps with TBS-T, membranes were probed with an anti-rabbit-antibody conjugated to horseradish peroxidase, and proteins were detected as described above.

### Western blotting of pSTAT3

Ba/F3-gp130-IL-6R and Ba/F3-gp130-IL-6RΔ S359_S361 cells were washed 3 times with 10 ml PBS. 3 wells per cell line of a 12-well plate were filled with 1 ml DMEM without supplements containing 1 ×  10^6^ cells. After 2 h of incubation at 37 °C cells were stimulated with 10 ng/ml IL-6 or Hyper IL-6 at 37 °C for 15 min. One control well per cell line remained untreated. Cells were harvested and the cell pellets were boiled in 50 μ l 2.5× Laemmli buffer at 95 °C for 10 min. 20 μ l of each sample were loaded on SDS-PAGE and blotted as described above. The membrane was blocked with 5% skim milk powder in TBS-T and probed with 1:1000 diluted anti phospho-STAT3 rabbit mAb (Cell Signaling) at 4 °C overnight. After 5 washing steps with TBS-T, membranes were probed with anti-rabbit-antibody conjugated to horseradish peroxidase diluted in 5% skim milk powder in TBS-T. Proteins were detected with West Pico Substrate Western Blotting Detection Kit (Thermo Scientific, Waltham, MA, USA) according to the manufacturer’s instructions.

Before probing for STAT3 as loading control, membranes were stripped with stripping buffer (62.5 mM Tris-HCl, pH 6.8, 2% SDS, 0.1% β -mercaptoethanol) for 30 min at 60 °C, washed 5 times, and blocked again. The membranes were probed with anti STAT3 mouse mAb (Cell Signaling, Cambridge, UK) and an anti-mouse-antibody conjugated to horseradish peroxidase and developed as describes above.

### Flow cytometry

For detection of cell surface expression, 1 ×  10^6^ of the respective Ba/F3-gp130-IL-6R cell lines or the transiently transfected HEK293 cells were washed 3 times with 1 ml FACS buffer (PBS, 0.5% BSA). Incubation was performed in 200 μ l FACS buffer containing 1:100 diluted anti-hIL-6R 4 –11 mAb (1 mg/ml) for 60 min on ice. After 3 washing steps with FACS buffer, cells were incubated in 200 μ l FACS buffer containing a 1:100 dilution of APC-conjugated anti-mouse mAb (Dianova, Hamburg, Germany). Cells were washed 3 times with FACS buffer, suspended in 500 μ l FACS buffer, and analyzed by flow cytometry on a BD Biosciences FACS Canto and FCS Express V3 (De Novo Software, Los Angeles, CA).

### Enzyme-linked ImmunoSorbent Assay

The ELISA which specifically detects soluble as well as full length human IL-6R was described previously^38^, and performed with streptavidin-horseradish peroxidase (R&D Systems, Minneapolis, MN, USA) and the peroxidase substrate BM blue POD (Roche, Mannheim, Germany). The enzymatic reaction was stopped by addition of 1.8 M sulfuric acid and the absorbance read at 450 nm on a Tecan rainbow reader (Tecan, Crailsheim, Germany).

### Cell viability assay

Proliferation of the different Ba/F3-gp130-IL-6R cell lines in response to IL-6 and Hyper-IL-6 was determined using the Cell Titer Blue Cell viability assay reagent (Promega, Karlsruhe, Germany) following the manufacturer’s protocol as described previously^43^. Values were measured in triplicates per experiment, and relative light units (RLU) obtained after 60 min were normalized by subtraction of control values obtained after 0 min.

### Model building of the ADAM17 - IL-6R complex

The model structure of the human ADAM17 catalytic domain in complex with the corresponding stalk region of the human IL-6R was build using the X-ray structure of the ADAM17/TIMP3 complex as a template (pdb accession code: 3cki)^45^. The seven N-terminal amino-acid residues of TIMP3 (CTCSPSH) are bound in the groove constituting the P’ side of the catalytic domain and were exchanged to the corresponding amino-acid residues of the human IL-6R stalk region (QDSSSVP or PVQDSSS). Amino-acid residues were exchanged using a database-search approach included in the software package WHATIF^46^.

### Presentation of experimental data

Data are expressed as mean values ±  standard deviation throughout the manuscript. ELISA data are derived from three independent experiments. For flow cytometry, Western blots and cell viability assays one from at least three experiments with similar outcome is shown.

## Additional Information

**How to cite this article**: Riethmueller, S. *et al.* Cleavage Site Localization Differentially Controls Interleukin-6 Receptor Proteolysis by ADAM10 and ADAM17. *Sci. Rep.* **6**, 25550; doi: 10.1038/srep25550 (2016).
